# Expression of Molecular Differentiation Markers Does Not Correlate with Histological Differentiation Grade in Intrahepatic Cholangiocarcinoma

**DOI:** 10.1371/journal.pone.0157140

**Published:** 2016-06-09

**Authors:** Céline Demarez, Catherine Hubert, Christine Sempoux, Frédéric P. Lemaigre

**Affiliations:** 1 de Duve Institute, Université catholique de Louvain, Avenue Hippocrate 75, B-1200 Brussels, Belgium; 2 Division of Hepato-Biliary and Pancreatic Surgery, Department of Abdominal Surgery and Transplantation, Cliniques universitaires Saint-Luc, Université catholique de Louvain, Avenue Hippocrate 10, B-1200 Brussels, Belgium; 3 Service de Pathologie Clinique, Centre Hospitalier Universitaire Vaudois, Université de Lausanne, CH-1011 Lausanne, Switzerland; Digestive Disease Research Center, Scott & White Healthcare, UNITED STATES

## Abstract

The differentiation status of tumor cells, defined by histomorphological criteria, is a prognostic factor for survival of patients affected with intrahepatic cholangiocarcinoma (ICC). To strengthen the value of morphological differentiation criteria, we wished to correlate histopathological differentiation grade with expression of molecular biliary differentiation markers and of microRNAs previously shown to be dysregulated in ICC. We analysed a series of tumors that were histologically classified as well, moderately or poorly differentiated, and investigated the expression of cytokeratin 7, 19 and 903 (*CK7*, *CK19*, *CK903*), SRY-related HMG box transcription factors 4 and 9 (*SOX4*, *SOX9*), osteopontin (*OPN*), Hepatocyte Nuclear Factor-1 beta (*HNF1β*), Yes-associated protein (*YAP*), Epithelial cell adhesion molecule (*EPCAM*), Mucin 1 (*MUC1*) and N-cadherin (*NCAD*) by qRT-PCR and immunostaining, and of *miR-31*, *miR-135b*, *miR-132*, *miR-200c*, *miR-221* and *miR-222*. Unexpectedly, except for subcellular location of SOX9 and OPN, no correlation was found between the expression levels of these molecular markers and histopathological differentiation grade. Therefore, our data point toward necessary caution when investigating the evolution and prognosis of ICC on the basis of cell differentiation criteria.

## Introduction

Intrahepatic cholangiocarcinoma (ICC) is the second most common primary tumor of the liver. It represents less than 10% of cholangiocarcinoma cases, but in contrast to perihilar and distal cholangiocarcinoma, its incidence and mortality rates are rising [1–3]. When evaluating disease progression, several prognostic factors were proposed. These include tumor size, surgical strategy, serum markers, TNM staging, histological features, mutational profiles, gene expression signatures (mRNA, microRNA, lncRNA), or expression of individual genes at the mRNA, miRNA or protein level. Prognostic factors can also be evaluated combinatorially, for instance in the format of nomograms, to improve their prognostic value [4].

Because of its ease of use in the clinic, differentiation of ICC is an often considered prognostic factor. Histopathologically, the proportion of gland formation serves as a differentiation criterium: well-differentiated tumors, which are associated with better survival, exhibit greater than 95% glandular tissue, moderately differentiated tumors show 50% to 95% gland formation, and poorly differentiated tumors have less than 50% glandular tissue [5–8]. Interestingly, a number of markers that qualify as molecular differentiation markers because they were shown to promote differentiation of cholangiocytes or to be expressed specifically in the biliary lineage correspond to ICC prognostic factors. One would therefore expect that expression of molecular differentiation markers would overlap with the prognostic value of ICC. Consistently, Mazur and coworkers found that the transcription factor SRY-related HMG box transcription factor 9 (*SOX9*), which is required for bile duct morphogenesis and cholangiocyte polarity, is a prognostic factor for survival [7]. Moreover, Terashi *et al*. showed that lack of osteopontin (*OPN*), which is expressed in the cholangiocytes and not in hepatocytes [9], is associated with poor patient outcome [10]. However, *SOX9* was not confirmed as a predictor of better survival in a different study [11], and not all molecular differentiation markers have a prognostic value: *HNF1β*, which is cholangiocyte-specific in adult liver and which stimulates biliary development [12], is not associated with survival rate of ICC [7, 13]. Again, Wang and coworkers showed that the transcription factor *SOX4*, which promotes cholangiocyte differentiation and bile duct formation [14], is unexpectedly a factor for poor prognosis of ICC [15].

These observations shed light on a discrepancy between the prognostic value and the expression of molecular differentiation markers and prompted us to consider that the expression of molecular markers of cholangiocyte differentiation would have a stronger predicting value if supported by histopathological differentiation criteria. Here we analysed a cohort of surgically resected ICCs and verified if histopathological differentiation grade is correlated with expression of molecular differentiation markers. For the latter, our analysis included transcription factors that are well characterized as differentiation regulators. We also included a set of microRNAs (miRNAs) which have been shown to be differentially expressed between the different tumor grades [8]. Indeed, miRNAs are involved in cholangiocarcinoma where they modulate apoptosis, proliferation, migration and response to therapy [16–19] and several investigators characterized miRNA expression profiles in ICC to identify miRNA signatures for improved diagnosis and prognosis [20–24].

Surprisingly, we found that molecular differentiation markers are expressed at similar levels regardless of the differentiation status of the tumor. Therefore, our data highlight a disparity between molecular- and histology-based classification and suggest caution when classifying ICC tumor samples.

## Materials and Methods

### Ethics Statement

The work on human tissue samples was performed in compliance with the Belgian regulation, and with the 1975 Declaration of Helsinki. We studied 18 ICCs obtained from 16 patients (12 men; 4 women) surgically treated at the Cliniques Universitaires St Luc (Brussels, Belgium) by a surgeon who is not co-author of the present paper, prior to the start of the present analysis. An anonymous registration number was tagged on each sample and researchers who analysed the samples had no access to the name of the donor. The retrospective analysis of the surgical specimens was approved by the Commission on Biomedical Ethics of the Université Catholique de Louvain and Cliniques Universitaires Saint-Luc (Approval # 2013/08JUL/384). Informed consent is not required for retrospective analyses.

### Immunohistochemistry and Immunofluorescence

Immunohistochemistry for cytokeratin (CK) 7, CK19 and H&E stainings were performed on 4 μm sections of formalin-fixed paraffin-embedded tumors and carried out with a standardized protocol on a Ventana Benchmark system (Ventana Medical Systems, Tucson, AZ, USA). Immunofluorescence was performed on 5 μm sections of formalin-fixed paraffin-embedded tumors. Briefly, tissue sections were deparaffinized and micro-wave heated for 10 min in 10 mM sodium citrate pH 6.0 for antigen unmasking. Sections were permeabilized for 5 min in phosphate-buffered saline (PBS)/0.3% Triton X-100 before blocking for 45 min in 0.3% milk/10% bovine serum albumin/0.3% Triton X-100 in PBS. Primary and secondary antibodies (listed in S1 Table) were diluted in blocking solution and incubated respectively at 4°C overnight and room temperature for 1 h. Pictures were taken with an Axiovert 200 fluorescent microscope using AxioVision system.

### RNA extraction and RT-qPCR

Formalin-fixed paraffin-embedded tumors were sectioned at 10 μm and regions of interest were scraped from the glass slides with a surgical blade. Total RNA was isolated using the RecoverAll Total Nucleic Acid Isolation Kit for FFPE (#AM1975, Ambion). The procedure included a crosslink reversal step during RNA isolation and the use of random hexamers and short amplicon size for the RT-qPCR step [25]. cDNA synthesis was performed with MMLV reverse transcriptase (#28025–13, Invitrogen) according to manufacturer’s protocol. Gene expression was quantified by qPCR using Kapa SYBR Fast 2X Universal Master Mix (#KK4601, Sopachem). The primers are listed in S2 Table. For measuring microRNA expression, specific stem-loop primers were used for reverse transcription and RT-qPCR was performed using a specific forward primer and a common universal reverse primer (S3 Table). mRNA and microRNA levels were normalized for *β-ACTIN* and for means of *RNU6* and *RNU1a* respectively. mRNA and miRNA could be compared between tumor and non-tumor samples: mean Ct values for *β-ACTIN* were 20.21 and 21.70 in tumor and non-tumor, respectively; mean Ct values for means of U6 and U1A were 15.19 and 16.37 in tumor and non-tumor tissue.

## Results

### Selection of tumor samples

**Fig 1:**
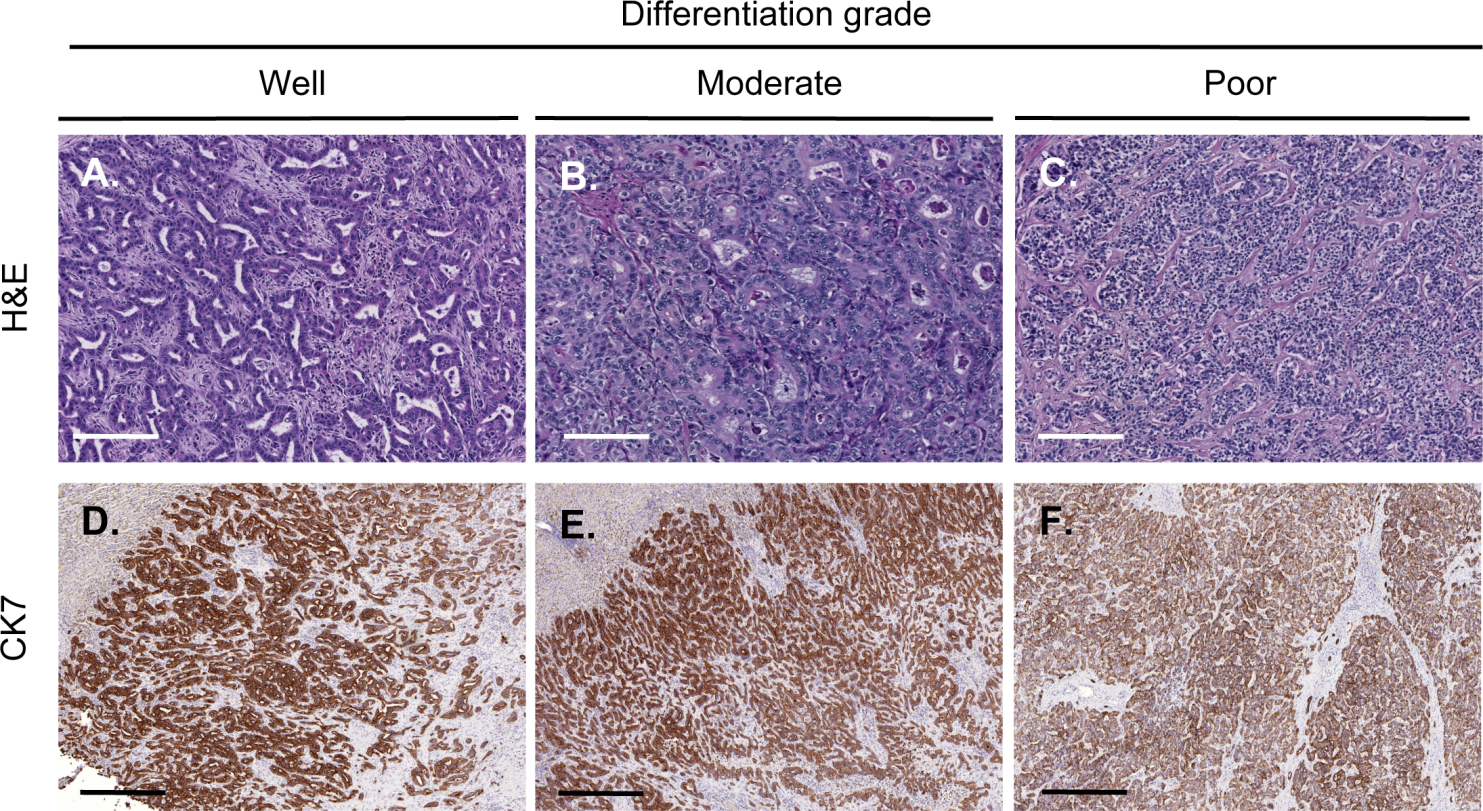
Tumor classification and selection of tumor areas with high density of *CK7*+ cells. **(A-C)** Tumors were classified as well-, moderately- and poorly-differentiated. **(D-F)** Immunohistochemical detection of *CK7* in representative histologically-graded ICCs. Areas with at least 50% of *CK7* staining were selected for further RNA extraction and immunofluorescence stainings. White scale bars = 200 μm. Black scale bars = 500 μm

We histologically classified 18 ICC samples from 16 patients (12 men and 4 women; age 39–75, mean: 60.2) as well (containing 95% glandular tissue), moderately (50% to 95% gland formation) or poorly differentiated (less than 50% glandular tissue) (Fig 1A–1C). None of the patients were receiving treatment, except for one of the two patients with a recurrence and who was treated with Gemcitabine. In one patient, the ICC contained both a well and a poorly differentiated area that were analysed separately. Two patients experienced a recurrence and each second tumor was also studied separately, leading to a total number of 19 samples of ICC characterized for their histological differentiation. We selected tumors areas in which *CK7*-positive cells represented more than 50% of the tumor surface (Fig 1D–1F), in order to reduce the contamination of epithelial cancer cells by non-epithelial cells during the RNA extraction procedure (see below). Tumor characteristics are described in Table 1.

**Table 1:** Characteristics of tumors samples.

ICC	
Size	2–16 cm (mean = 5.9)
Macroscopic type	17 mass forming
	1 periductal infiltrative
Microscopic differentiation	5 well-differentiated
	6 moderately differentiated
	6 poorly differentiated
	1 with both well and poorly differentiated area
Mucin production	5 present
	13 absent
Background liver	3 with cirrhosis
	15 without cirrhosis
Pathological stage	4 pT1N0; 2 pT1N1
	6 pT2aN0; 1 pT2aN1
	2 pT2bN0; 2 pT2bN1
	1 pT4N1

### Molecular differentiation markers in ICC: mRNA and microRNA expression

**Fig 2:**
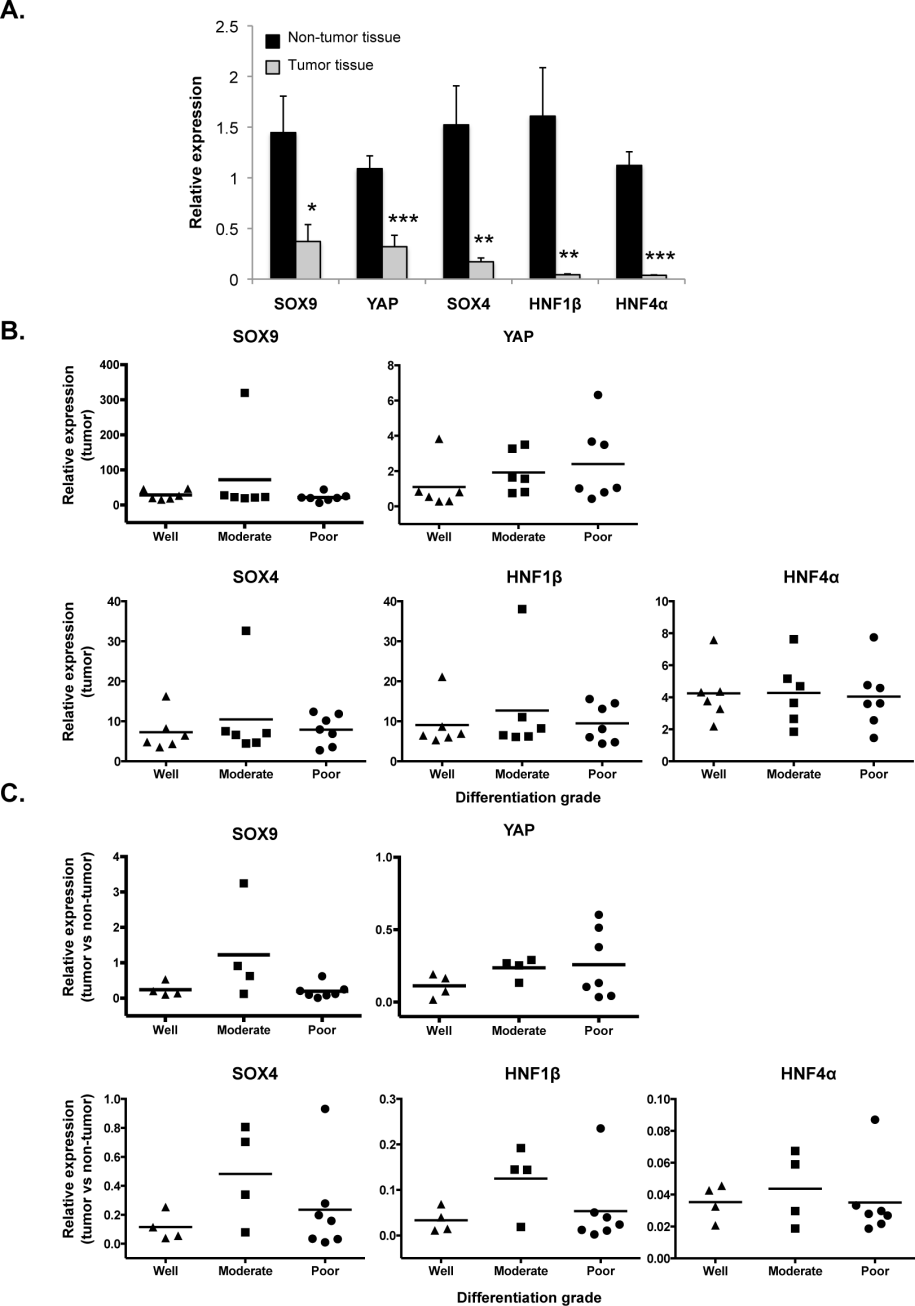
Molecular biliary differentiation markers are not differentially expressed among histologically-graded iCC's. **(A)** All biliary differentiation markers are expressed (qRT-PCR) at lower levels in ICC as compared to distant non-tumoral tissue. Low levels of *HNF4α* indicate lack of significant contamination with normal hepatocytes. (Mean ± SEM; *p<0.05, **p<0.01, ***p<0.001). **(B-C)** Lack of correlation between molecular differentiation marker expression and tumor grade when comparing **(B)** ICC samples or **(C)** the ratio of expression in tumor versus non-tumor tissue. Dots represent individual ICC samples and horizontal bars represent the mean of the samples.

To look for a potential correlation between histopathological differentiation grade and molecular differentiation markers in ICC, we measured the expression of the latter in the tumor samples. We focused on transcription factors that were shown to be stimulators of biliary differentiation. Total RNA was extracted from formalin-fixed paraffin-embedded tissue: ICC tissue was delineated using *CK7* and H&E staining in the adjacent sections, and non-tumor *CK7*-negative tissue from a distant sample of the same surgical specimen was used as control. We then measured by qRT-PCR the expression of biliary differentiation markers (*SOX4*, *SOX9*, *and HNF1β*) and of the hepatocyte marker *HNF4α*. All biliary markers were expressed at lower levels in the tumor tissue than in the adjacent non-tumor tissue (Fig 2A). *HNF4α* was low, confirming that the tumor samples were not contaminated with significant levels of hepatocytes.

Importantly, our analysis showed that none of the tested markers correlated with histological differentiation grade (Fig 2B). In addition, when the ratio of expression in tumor *versus* non-tumor tissue was analysed, again no correlation was found between molecular marker expression and histological grade of the ICCs (Fig 2C).

**Fig 3:**
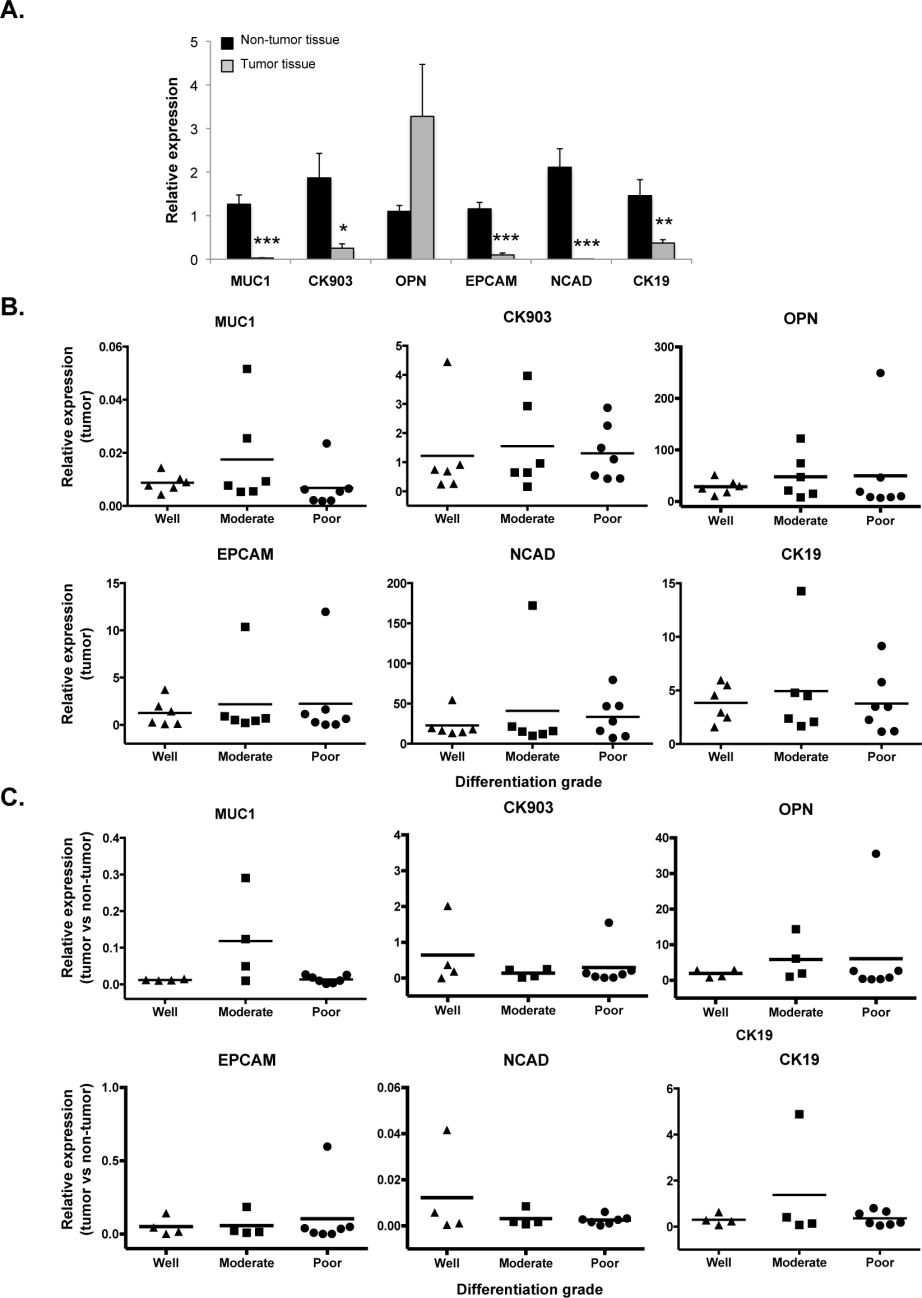
Biliary-specific proteins are not differentially expressed among histologically-graded ICC's. **(A)** All markers are expressed (qRT-PCR) at lower levels in ICC as compared to distant non-tumoral tissue. (Mean ± SEM; *p<0.05, **p<0.01, ***p<0.001). **(B-C)** Lack of correlation between molecular differentiation marker expression and tumor grade when comparing **(B)** ICC samples or **(C)** the ratio of expression in tumor versus non-tumor tissue. Dots represent individual ICC samples and horizontal bars represent mean of the samples.

Since the transcription factors tested above did not correlate with ICC histological grade, we measured the expression of other markers (*OPN*, *MUC1*, *CK903*, *YAP*, *EPCAM*, *NCAD* and *CK19*) that are known to be biliary-specific and associated with ICC [26–32]. All those markers were expressed at lower levels in the tumor tissue than in the adjacent non-tumor tissue (Fig 3A). Moreover, as observed previously, we did not found any correlation between the biliary markers and the histological grade of the tumors (Fig 3B). Again, when considering the ratio of expression in tumor versus non-tumor tissue, no correlation was found (Fig 3C).

We next investigated the expression of miRNAs in our ICC samples. Several studies have compared the expression of miRNAs in ICC and control tissue [20–24]. We here analysed miRNAs that were found to be dysregulated in earlier studies in which control tissue was compared with ICC, and whose dysregulation has been correlated with histological differentiation grade of ICCs (Table 2) [8].

**Table 2:** Selection of microRNAs associated with ICC differentiation grade.

microRNA	Dysregulation in ICC	Differential expression between ICC histological-grades^[^^8^^]^
miR-200c	Up-^[^^8^^,^ ^33^^]^ or Down-^[^^21^^,^ ^23^^]^ regulated	Moderate > Well (FC = 9,6)
miR-221	Up-^[^^8^^,^ ^33^^]^ or Down-^[^^23^^]^ regulated	Moderate > Well (FC = 4,6)
miR-31	Up-^[^^8^^,^ ^33^^]^ or Down-^[^^23^^]^ regulated	Moderate > Well (FC = 60,3)
miR-222	Down-regulated^[^^21^^,^ ^23^^]^	Moderate > Well (FC = 4,8)
miR-135b	Up-regulated^[^^8^^,^ ^33^^]^	Moderate > Well (FC = 22,7)
miR-132	No	Moderate > Well (FC = 15,1)

FC: Fold Change

**Fig 4:**
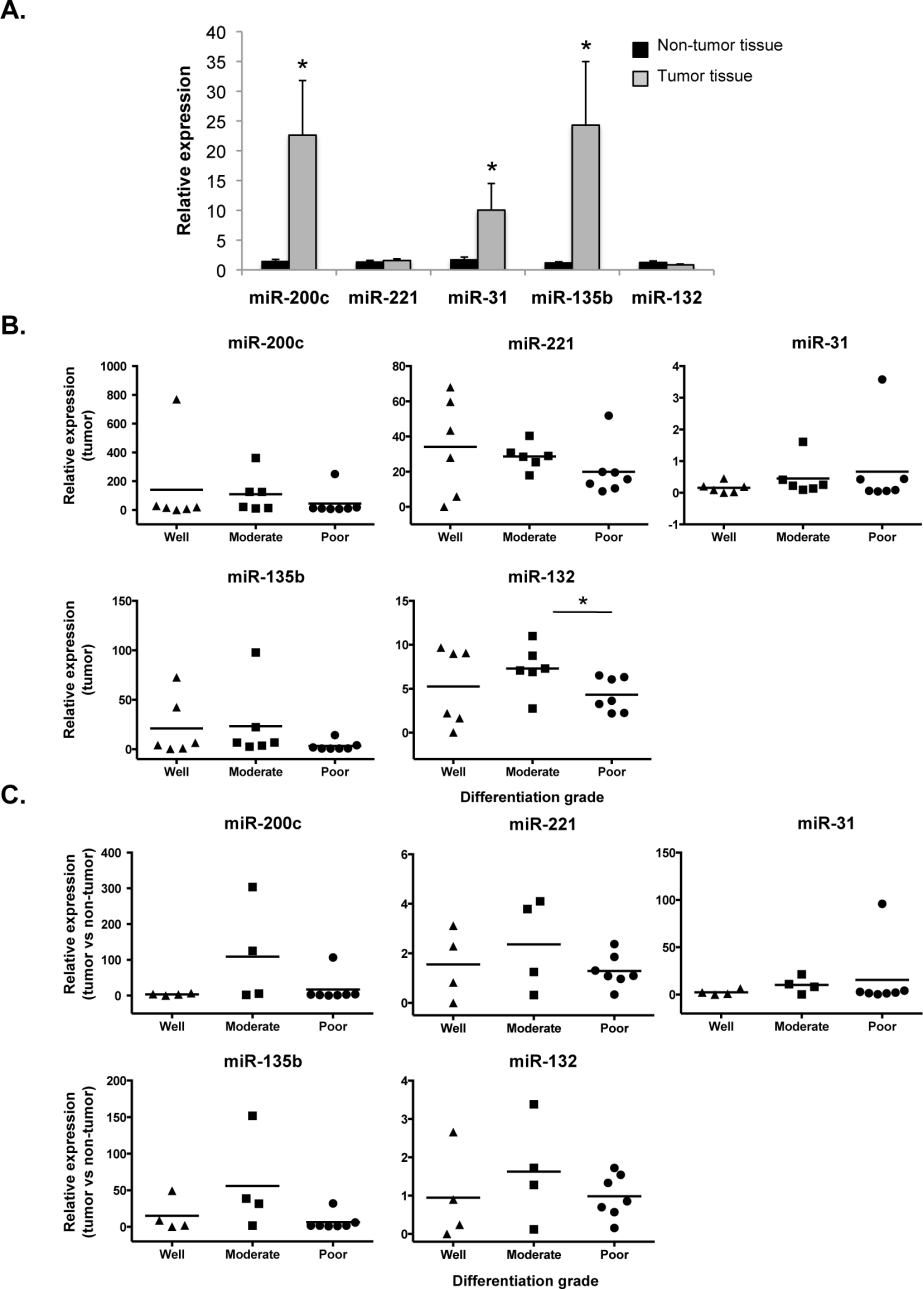
ICC-associated microRNAs are not differentially expressed in histologically-graded ICC. MicroRNAs known to be dysregulated in ICC and differentially expressed among tumor grades were selected for qRT-PCR analysis. **(A)** Three microRNAs are up-regulated in ICC when compared to distant non-tumoral tissues. (Mean ± SEM; *p<0.05). **(B-C)** Lack of correlation between microRNA expression and tumor differentiation grade when **(B)** comparing ICC samples, or **(C)** when analysing the ratio of expression in tumor *versus* non-tumor tissue. Dots represent individual ICC samples.

Fig 4A shows that a subset of microRNAs (*miR-200c*, *miR-31 and miR-135b*) was expressed at higher levels in the tumor than in the non-tumor tissue. *miR-222* was not detectable in our ICC samples. When considering differentiation of the tumors, no correlation was found between histological grade and expression of the selected miRNAs, except for *miR-132*, which was low in the poorly differentiated tumors (Fig 4B). However, when considering the ratio of expression in tumor *versus* non-tumor tissue, again no correlation was found with histological grade, even for *miR-132* (Fig 4C).

### Molecular differentiation markers in ICC—protein expression

**Fig 5:**
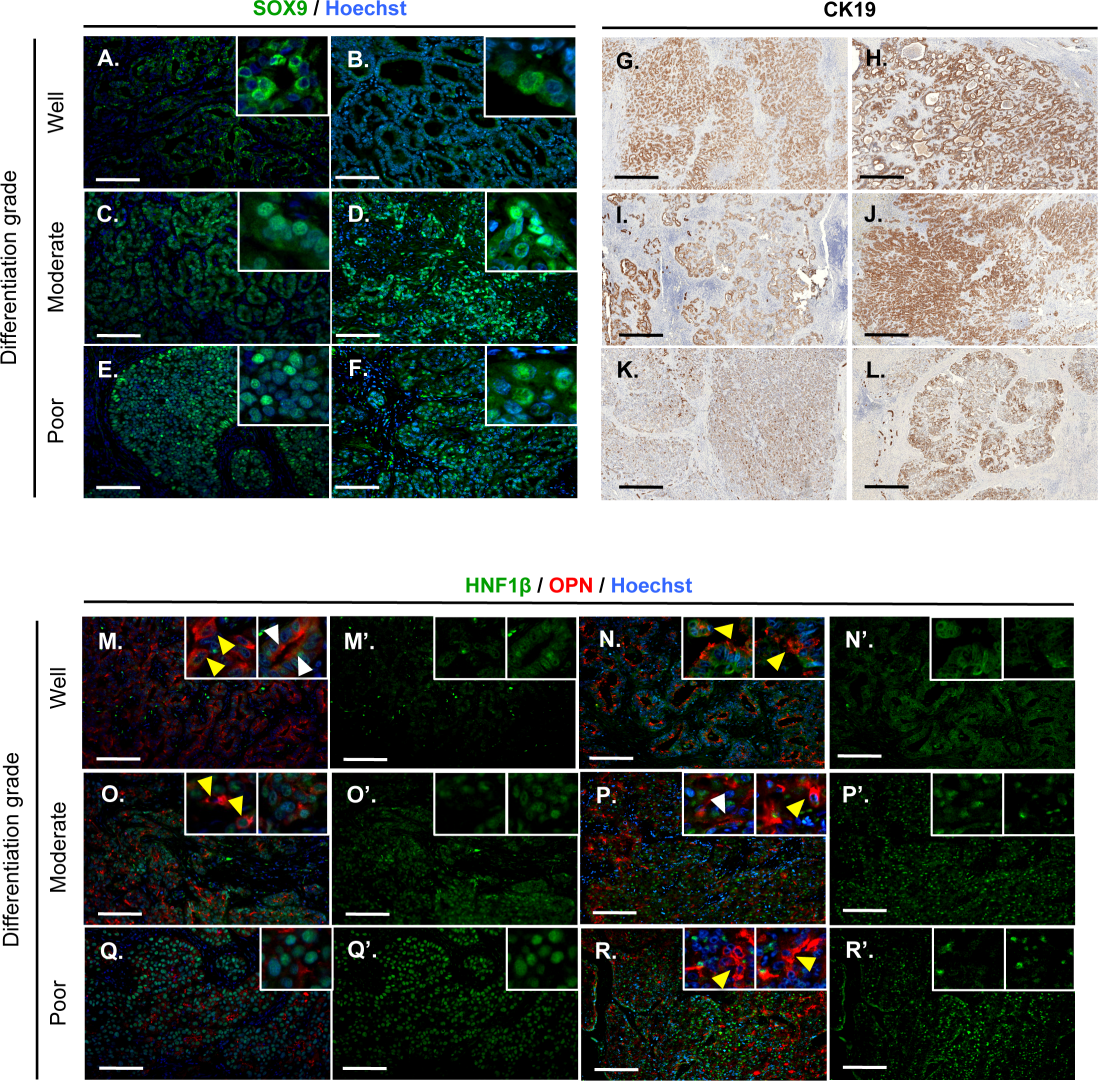
Expression of molecular biliary markers in histologically-graded ICC's. **(A-F)** SOX9 expression is detected in the cytoplasm of well-differentiated ICC cells (A-B) and both in the cytoplasm and nucleus of moderately and poorly differentiated tumor cells (C-F). **(G-L)** CK19 is expressed in all types of histologically-graded tumors. **(M-R)** OPN is detected in all types of histologically graded-ICC's. Its expression is mostly apical in well-differentiated tumor cells (M-N, white arrowheads), but cytoplasmic OPN is detected in moderately and poorly differentiated ICCs and in a subset of well-differentiated tumor cells (M-R, yellow arrowheads). **(M’-R’)** HNF1β expression inversely correlates with ICC differentiation grade. White scale bar = 100 μm, black scale bar = 500 μm.

Since mRNA expression does not necessarily correlate with protein expression, we analysed the expression of SOX9, OPN, CK19 and HNF1β by immunostaining on two representative samples of each histologically-defined differentiation grade (Fig 5). The results showed that the markers are expressed in the tumors regardless of their differentiation grade. SOX9, which is normally located in the nucleus, was restricted to the cytoplasm in well-differentiated ICC while it was expressed in both the cytoplasm and nucleus in moderately and poorly differentiated ICC (Fig 5A–5F). CK19 was expressed in all ICC samples (Fig 5G–5L). Similarly, OPN was expressed in all tumor types (Fig 5M–5R). However, in well-differentiated ICC, OPN was predominantly expressed at the apical pole of cells but a subset of cells also displayed cytoplasmic location (Fig 5M and 5N, white and yellow arrowheads). In contrast, OPN was observed mainly in the cytoplasm of cells of moderately differentiated tumors, while only a subset of cells expressed OPN at the apical pole (Fig 5O and 5P, white and yellow arrowheads). Poorly differentiated tumors have lost apico-basal polarity, preventing correct interpretation of polarity marker location. However, in those poorly differentiated tumors some cells expressed cytoplasmic OPN (Fig 5Q and 5R, yellow arrowheads). Therefore, overall expression of CK19, SOX9 and OPN protein does not correlate with histological differentiation grade, but subcellular location of SOX9 and OPN were related to ICC cell differentiation.

Surprisingly, in contrast to the qRT-PCR data, HNF1β protein expression was high in poorly differentiated ICC (Fig 5Q–5R and 5Q'–5R') and, low and mislocated, *i*.*e*. non-nuclear, in well differentiated tumors (Fig 5M–5N and 5M'–5N'); moderately differentiated tumors showed intermediate expression levels (Fig 5O–5P and 5O'–5P').

## Discussion

In recent years, many efforts have been devoted to better classify ICCs and determine their prognosis. Since differentiation grade of the tumors, defined by histopathological criteria, is closely related to patient outcome [6, 7], in the present work we wished to strengthen histopathological differentiation criteria by correlating them with expression of molecular cholangiocyte differentiation markers. We assumed that well differentiated tumors would express high levels of cholangiocyte markers and that these levels would decrease in less differentiated tumors. However, our work unexpectedly revealed a lack of correlation between histopathological and molecular differentiation criteria in ICC.

Here, we analysed the expression of biliary markers in areas of ICCs where the tumoral cells (highlighted by the CK7 positive staining) represented more than 50% of the total area in order to limit contaminations by non-tumor cells. All our carcinoma express low levels of these markers irrespective of the tumor differentiation grade (Figs 2 to 4), thereby suggesting that the epithelial cells are undergoing a dedifferentiation process, at least at the molecular level. In line with this observation, Mazur and coworkers previously proposed that *SOX9* expression decreases in the early step of ICC development [7]. These results raise the question of the real differentiation status of the tumors, and may explain why some differentiation markers are associated with good or poor prognosis in distinct studies (e.g. *SOX9*; [7, 11]), and why some other differentiation markers such as HNF1β are not associated with prognosis [7, 13].

We further showed by RT-qPCR and immunofluorescence that *SOX9* and *OPN* are expressed equally in all tumor types (Fig 5). However, their subcellular location depended on the differentiation grade of the ICC. SOX9 is located in the cytoplasm of well-differentiated tumors investigated here, whereas it was nuclear or cytoplasmic in moderately and poorly differentiated tumors. Cytoplasmic expression of SOX9 has already been observed in pancreatic adenocarcinoma affected with p53 mutation [34], in breast cancer cells [35] and in biliary tract cancers [7], suggesting that the location of SOX9 may depend on the mutational status of the cells. OPN is expressed in the cytoplasm of early biliary progenitors in the embryo, and becomes apical once the biliary cells become polarized [9]. The dual cytoplasmic and apical location of OPN found in well-differentiated tumors may reflect distinct levels of molecular differentiation of the cells within the same tumor. Regarding HNF1β, Mazur and coworkers, could not correlate HNF1β expression with survival rates of ICC [7]. This is at first sight at odds with our findings: since we found more HNF1β protein in poorly differentiated tumors, which are known to be associated with poor prognosis, our data would suggest that HNF1β is a marker of poor prognosis. However, combining our data with those of Mazur and coworkers would be in agreement with the overall conclusion of the present work, namely that molecular differentiation markers do not correlate with histopathological differentiation criteria. Finally, HNF1β protein expression is higher in poorly differentiated tumors, while *HNF1β* mRNA does not correlate differentiation grade. This points toward potential post-transcriptional regulation and further underscores the care needed when interpreting expression of molecular differentiation markers in ICC.

When analysing our set of tumors, we were unable to corroborate the findings by Plieskatt and co-workers, who showed that *miR-200c*, *miR221*, *miR-31*, *miR-135b* and *miR-132* are differentially expressed between well differentiated, moderately differentiated and papillary carcinoma-type ICC [8, 33]. In line with this, several groups attempted to list dysregulated microRNAs in ICCs, but only little overlap can be found when comparing the proposed microRNA lists [8, 21–24, 33]. In addition, contradictory results were found. For instance, *miR-200c* was either down-regulated [21, 23] or upregulated [8, 33] (Fig 3). This points towards necessary care when interpreting microRNA expression in ICC. The broad range of control tissue used to investigate microRNA expression could contribute to the observed discrepancies. Indeed, control tissues selected by different authors were either normal hepatic bile duct tissue or total liver parenchyma, which are distinct tissues, and originated from liver associated with a variety of diseases such as cirrhosis, hepatitis, or infection with *Opisthorchis viverrini* [8, 33, 36]. In that context, Plieskatt and coworkers showed that in *Opisthorchis viverrini*-induced ICC, miRNAs in distal non-tumor tissue clustered closer to the tumor than to non-tumor tissue from control individuals [33]. This is in line with our lack of correlation between miRNA or mRNA expression and ICC grade when considering the ratio of expression in tumor *versus* non-tumor tissue (Fig 3C). Indeed, our controls were from non-tumor tissue taken from the patient affected with ICC.

## Conclusion

In this work we compared histologically-graded ICC rich in epithelial cells and found no correlation between expression of biliary molecular differentiation markers and the histological differentiation grade of the tumors. Therefore, our data point towards necessary caution when classifying ICC tumor samples and determining prognosis based on differentiation criteria.

## Supporting Information

S1 Table List of primary and secondary antibodies used for immunostaining experiments. (PDF)

S2 Table List of primer sequences used for RT-qPCR. (PDF)

S3 Table List of stem-loop primers used for reverse transcription of miRNAs. (PDF)
