# Supplementary material for: Expression of Molecular Differentiation Markers Does Not Correlate with Histological Differentiation Grade in Intrahepatic Cholangiocarcinoma
Source: PLoS One. 2016 Jun 9;11(6):e0157140. doi: 10.1371/journal.pone.0157140 (PMC4900546; doi:10.1371/journal.pone.0157140)
Supplement: S1 Table — (PDF) [file pone.0157140.s001.pdf]

Supplementary Table 1: List of primary and secondary antibodies used for immunostaining experiments.

**Primary antibody**	**Species**	**Dilution**	**Source**	**Reference number**
CK7	Mouse	1/200	Dako	M7018
CK19	Mouse	1/200	NovoCastra	b170
Sox9	Rabbit	1/250	Chemicon	AB5535
HNF1β	Rabbit	1/100	Santa Cruz	SC-22840
OPN	Goat	1/150	R&D Systems	AF808
**Secondary antibody**	**Species**	**Dilution**	**Source**	**Reference number**
Alexa Fluor 488 anti-rabbit	Donkey	1/1000	Invitrogen	A-21206
Alexa Fluor 594 anti-goat	Donkey	1/1000	Invitrogen	A-11058
Alexa Fluor 647 anti-mouse	Donkey	1/1000	Invitrogen	A-31571
