# Supplementary material for: Expression of Molecular Differentiation Markers Does Not Correlate with Histological Differentiation Grade in Intrahepatic Cholangiocarcinoma
Source: PLoS One. 2016 Jun 9;11(6):e0157140. doi: 10.1371/journal.pone.0157140 (PMC4900546; doi:10.1371/journal.pone.0157140)
Supplement: S2 Table — (PDF) [file pone.0157140.s002.pdf]

Supplementary Table 2: List of primer sequences used for RT-qPCR.

Gene	Primer sequence (5' → 3')
β-Actin	Fw - TCC TGA GCG CAA GTA CTC TGT<br>Rev - CTG ATC CAC ATC TGC TGG AAG
Gapdh	Fw - GGC CAA GGT CAT CCA TGA CA<br>Rev - TTC TAG ACG GCA GGT CAG GT
RNU6	Fw – CTC GCT TCG GCA GCA CA<br>Rev – AAC GCT TCA CGA ATT TGC GT
RNU1a	Fw – CCA TGA TCA CGA AGG TGG TT<br>Rev – ATC CGG AGT GCA ATG GAT AA
SOX9	Fw - CCT GGG ATT GCC CCG A<br>Rev - GAG CAG ACG CAC ATC TC
OPN	Fw – GCA ACC GAA GTT TTC ACT CC<br>Rev – ATT CAA CTC CTC GCT TTC CA
CK19	Fw – CTG AAG GAA GAG CTG GCC TA<br>Rev – TCA TAT TGG CTT CGC ATG TC
SOX4	Fw – GGT GCA AGA CCC CGA GTG<br>Rev – GCT GTC TTT GAG CAG CTT CC
HNF4α	Fw – CGT GCT GCT CCT AGG CAA<br>Rev – GTC AAG GAT GCG TAT GGA CAC
HNF1β	Fw – ACA CAC CTC CCA TCC TCA AG<br>Rev – CGC TTC TGG GTC TTC ATA GG
MUC1	Fw – TGA GCG AGT ACC CCA CCT AC<br>Rev – CCC CTA CAA GTT GGC AGA AG
YAP	Fw –TAG CCC TGC GTA GCC GTT A<br>Rev – TCA TGC TTA GTC CAC TGT CTG T
CK903	Fw – CATGGGAGACAGCAAACCCT<br>Rev – TGCAGGACATCCATCTGCAA
EPCAM	Fw – GAA GGC TGA GAT AAA GGA GAT GGG<br>Rev – TTA ACG ATG GAG TCC AAG TTC TGG
NCAD	Fw – TGGATGGGCTGCCTCCAGGTGAC<br>Rev – ACCAGCCCACCCCTCGAGCCC
miR-200c	Fw – ACA CTC CAG CTG GGT AAT ACT GCC GGG TAA<br>Rev - CTC AAC TGG TGT CGT GGA GTC GGC AAT TCA GTT GAG TCC ATC ATT
miR-221	Fw – ACA CTC CAG CTG GGA GCT ACA TTG TCT GC<br>Rev – CTC AAC TGG TGT CGT GGA GTC GGC AAT TCA GTT GAG GAA ACC CA
miR-31	Fw – ACA CTC CAG CTG GGA GGC AAG ATG CTG GCA<br>Rev – CTC AAC TGG TGT CGT GGA GTC GGC AAT TCA GTT GAG CAG CTA TG
miR-135b	Fw – ACA CTC CAG CTG GGT ATG GCT TTT CAT TCC<br>Rev – CTC AAC TGG TGT CGT GGA GTC GGC AAT TCA GTT GAG TCA CAT AGG
miR-132	Fw – ACA CTC CAG CTG GGT AAC AGT CTA CAG CCA<br>Rev – CTC AAC TGG TGT CGT GGA GTC GGC AAT TCA GTT GAG CGA CCA TG
