# Supplementary material for: Expression of Molecular Differentiation Markers Does Not Correlate with Histological Differentiation Grade in Intrahepatic Cholangiocarcinoma
Source: PLoS One. 2016 Jun 9;11(6):e0157140. doi: 10.1371/journal.pone.0157140 (PMC4900546; doi:10.1371/journal.pone.0157140)
Supplement: S3 Table — (PDF) [file pone.0157140.s003.pdf]

Supplementary Table 3: List of stem-loop primers used for reverse transcription of miRNAs.

Gene	Primer sequence (5' → 3')
miR-200c	Rev - CTC AAC TGG TGT CGT GGA GTC GGC AAT TCA GTT GAG TCC ATC ATT
miR-221	Rev – CTC AAC TGG TGT CGT GGA GTC GGC AAT TCA GTT GAG GAA ACC CA
miR-31	Rev – CTC AAC TGG TGT CGT GGA GTC GGC AAT TCA GTT GAG CAG CTA TG
miR-135b	Rev – CTC AAC TGG TGT CGT GGA GTC GGC AAT TCA GTT GAG TCA CAT AGG
miR-132	Rev – CTC AAC TGG TGT CGT GGA GTC GGC AAT TCA GTT GAG CGA CCA TG
